# Putative small RNAs controlling detoxification of industrial cyanide-containing wastewaters by *Pseudomonas pseudoalcaligenes* CECT5344

**DOI:** 10.1371/journal.pone.0212032

**Published:** 2019-02-08

**Authors:** Alfonso Olaya-Abril, Víctor Manuel Luque-Almagro, María Dolores Pérez, Cristina María López, Francisco Amil, Purificación Cabello, Lara Paloma Sáez, Conrado Moreno-Vivián, María Dolores Roldán

**Affiliations:** 1 Departamento de Bioquímica y Biología Molecular, Edificio Severo Ochoa, Campus de Rabanales, Universidad de Córdoba, Córdoba, Spain; 2 Servicio Central de Apoyo a la Investigación (SCAI), Unidad de Proteómica, Campus de Rabanales, Córdoba, Spain; 3 Departamento de Botánica, Ecología y Fisiología Vegetal, Edificio Celestino Mutis, Campus de Rabanales, Universidad de Córdoba, Córdoba, Spain; Laurentian University, CANADA

## Abstract

The alkaliphilic bacterium *Pseudomonas pseudoalcaligenes* CECT5344 uses free cyanide and several metal−cyanide complexes as the sole nitrogen source and tolerates high concentrations of metals like copper, zinc and iron, which are present in the jewelry wastewaters. To understand deeply the regulatory mechanisms involved in the transcriptional regulation of cyanide-containing wastewaters detoxification by *P*. *pseudoalcaligenes* CECT5344, RNA-Seq has been performed from cells cultured with a cyanide-containing jewelry wastewater, sodium cyanide or ammonium chloride as the sole nitrogen source. Small RNAs (sRNAs) that may have potential regulatory functions under cyanotrophic conditions were identified. In total 20 sRNAs were identified to be differentially expressed when compared the jewelry residue *versus* ammonium as nitrogen source, 16 of which could be amplified successfully by RT-PCR. As predicted targets of these 16 sRNAs were several components of the *nit1C* gene cluster encoding the nitrilase NitC essential for cyanide assimilation, the *cioAB* gene cluster that codes for the cyanide-insensitive cytochrome *bd*-type terminal oxidase, the medium length-polyhydroxyalkanoates (ml-PHAs) gene cluster, and gene clusters related with a global nitrogen limitation response like those coding for glutamine synthase and urease. Other targets were non-clustered genes (or their products) involved in metal resistance and iron acquisition, such as metal extrusion systems and the ferric uptake regulatory (Fur) protein, and a GntR-like regulatory family member probably involved in the regulation of the cyanide assimilation process in the strain CECT5344. Induction of genes targeted by sRNAs in the jewelry residue was demonstrated by qRT-PCR.

## Introduction

Cyanide is a natural compound that is produced by living organism with offensives or defensive purposes. Cyanogenic organisms that are capable to produce cyanide include bacteria, algae, fungi and plants [1,2]. Numerous genera of cyanogenic bacteria have been described like *Chromobacterium*, *Burkholderia* and *Pseudomonas*, and several of their genomes have been sequenced recently [3,4,5]. Cyanogenic bacteria produce cyanide through a synthase that is encoded by the *hcnABD* gene cluster. Recently, it has been described the application of these bacteria to the biorecovery of gold from electroplate wastewaters. In mining and metal handling industries, the extraction of gold and other precious metals is achieved by cyanide addition (cyanidation process). In this sense, cyanogenic bacteria constitute a source of cyanide for metal bioleaching, forming soluble metal–cyanide complexes from solid materials [6,7,8]. By contrast, only a few genera of bacteria have been described to metabolize cyanide through different oxidative, reductive, hydrolytic or substitution/transfer pathways [2,9,10,11,12], and/or organic nitriles, which are assimilated by nitrilases or nitrile hydratases [2,13]. Most cyanide-degrading microorganisms grow best around neutral pH, which could be an inconvenience to assimilate cyanide because at neutral pH cyanide evaporates as cyanhydric acid (pKa 9.3). Considering this fact, alkalophilic microorganisms should be required to remove cyanide efficiently from contaminated areas and wastewaters. These cyanide-containing residues need to be treated to reduce their toxicity, but physical-chemical treatments have not been demonstrated to be very efficient and, therefore bioremediation processes constitute a potent alternative to decontaminate industrial cyanide-containing residues [14,15,16].

Cyanide is produced at high concentrations by industrial activities, mainly related to jewelry and mining. Cyanide appears strongly bound to metals in these industrial residues, which usually also contains metals like iron, copper and zinc [9,17,18]. Complexes of cyanide with copper, nickel or zinc can be easily dissociated in dissolution, while complexes with iron or cobalt are very stable, with dissociation constants ranging from 10^−17^ to 10^−52^ M, although UV radiation may be efficiently applied to break these complexes [11]. Microorganisms are also able to degrade metal–cyanide complexes. Thus, a microbial consortium that use as nitrogen source the metal–cyanide complexes from metal electroplating activities, and several strains of *Pseudomonas* isolated from a mine of copper able to degrade free cyanide and metal–cyanide complexes have been described [18].

The alkaliphilic bacterium *Pseudomonas pseudoalcaligenes* CECT5344 uses cyanide or metal–cyanide complexes with zinc, copper and iron as the sole nitrogen source [9,19]. Recently, the whole genome of the strain CECT5344 has been sequenced and annotated [20,21,22]. This information has allowed the study of the global response to cyanide and metals present in the cyanide-containing jewelry wastewaters through DNA microarrays and quantitative proteomic analyses [23,24]. Among genes induced by cyanide in the strain CECT5344 were those coding for the alternative oxidase CioAB required for the cyanide insensitive respiration [25], the nitrilase NitC essential for cyanide assimilation [26] and other nitrilases, and several cellular components involved in different processes of biotechnological interest like furfural degradation and polyhydroxyalkanoate biosynthesis [9,27].

Small RNAs are usually non-coding transcripts of about 40–500 nucleotides in length that display regulatory properties [28,29,30]. Several scientific reports on bacteria have revealed that they can modulate many different metabolic pathways and stress responses [30,31]. The major families of sRNAs include antisense sRNAs that are encoded from the complementary strand to the target mRNA, sRNAs also encoded from the complementary strand to the target mRNA, but annealing with limited complementarity with the target, and sRNAs that bind to proteins affecting to enzymic activities [29].

In this work, putative sRNAs expressed by *P*. *pseudoalcaligenes* CECT5344 cells degrading the cyanide-containing jewelry wastewaters have been identified and subjected to bioinformatic analyses to obtain a holistic view of the sRNA molecules that may play a regulatory role during this biodegradative process. The presence of these sRNAs in cells grown with sodium cyanide highlights that sRNAs-mediated regulatory mechanisms may be involved in the regulation of cyanide-containing wastewaters detoxification by *P*. *pseudoalcaligenes* CECT5344.

## Materials and methods

### Chemicals

The industrial cyanide-containing wastewater from the jewelry was supplied by Avenir S.L. and Gemasur S.L. (Córdoba, Spain). Ammonium chloride, sodium cyanide and sodium acetate were supplied by Sigma–Aldrich (St. Louis-MO, USA). All other chemicals used in the study were of analytical grade. Solutions were prepared by using Milli-Q water (Millipore, Bedford-MA, USA). Wastes containing cyanide or other toxic chemicals were handled and disposed by the Environmental Protection Unit, University of Córdoba.

### Bacterial growth determination

Bacterial growth was determined by following the absorbance at 600 nm (A_600_) in a spectrophotometer.

### Bacterial culture conditions

To isolate *P*. *pseudoalcaligenes* CECT5344 RNAs, cells were cultured in 100 mL Erlenmeyer flasks filled to 20% with the phosphate-containing minimal medium M9 [32] at pH adjusted to 9.5. Sodium acetate (50 mM) and ammonium chloride (2 mM) were used as carbon and nitrogen sources, respectively. After 24 h, when ammonium was totally consumed, the specific nitrogen source sodium cyanide, cyanide from the jewelry wastewater or ammonium chloride was added to the media (2 mM concentration). Three independent biological replicates were carried out for each nitrogen source used. Cultures were kept at 30°C and continuous agitation at 220 rpm on a shaker. When cultures reached an absorbance of 0.3 at 600 nm and the remaining nitrogen source in the media was about 30–50%, 10 mL-aliquots were harvested by centrifugation at 4,000 × *g* for 15 min, as previously described [27]. Pellets were kept at -80°C until use.

### Analytical determinations

Ammonium concentration in the culture media was determined as described previously [33]. The concentration of free cyanide and weak metal–cyanide complexes was determined colorimetrically [34]. Total cyanide (free, weak metal–cyanide complexes and strong metal–cyanide complexes) was determined in a manifold system coupled to a peristaltic pump and a photodissociation reactor with a 20 W-UV lamp associated to a pervaporation cell. Additionally, a cyanide-selective electrode was used [24].

### Total RNA isolation

*P*. *pseudoalcaligenes* CECT5344 cells were suspended in 500 μl TRIsure^TM^ (Bioline International, Toronto, Canada) and total RNA was isolated using the Direct-zol^TM^ RNA Miniprep kit (Zymo Research, California, USA) according to the instruction of the manufacturer. DNase incubation was carried out in the column with RNase-free DNase (Zymo Research) and, when required, an additional post-column treatment was applied with DNase I (Ambion-Thermo Fisher Scientific, Masachusets, USA). Quality and quantity of the total RNAs were checked with Bioanalyzer (Agilent Technologies, Wilmington, DE, USA) and ND1000 spectrophotometer (Nanodrop 1000, Agilent Technologies). All samples showed an integrity number (RIN) higher than 6.9.

### Library preparation and sequencing

Library preparation and sequencing were performed by STABvida (Caparica, Portugal). After the integrity number (RIN) and concentration of RNAs were analyzed to ensure its sufficient integrity (> 6.9) and quantity (10 μg), cDNA was synthetized by using the Small RNA-Seq Library Preparation Kit (Bioo Scientific, Austin, Texas-USA). The resulting DNA library was sequenced in the Ilumina Hiseq 2500 platform using 100 pb single-end sequencing reads. The corresponding FASTQ files were obtained as result.

### Prediction and bioinformatic analysis of sRNAs

Raw reads were trimmed using Cutadapt 1.14. with ambiguous limit 2 nucleotides, quality limit 0.01 (error probability) and removal 5’- and 3’-ends 4 bp. The Rockhopper software [35] was used to predict unannotated RNA in *Pseudomonas pseudoalcaligenes* CECT5344 (HG916826.1) reference genome with strand specific considered, seed length 0.33, allowed mismatches 0.15 and minimum expression of UTR and ncRNAs 0.8. Reads aligned to unannotated regions (higher than 30% in all samples) were selected. Raw (fastaq files), processed data (wig files) and mapping (bam files) are available on the GEO database (Series record number: GSE117374).

High quality sequencing reads were mapped against the genome using a length fraction 0.95 and a similarity fraction 0.95 [36]. Resulting data were used to determine the expression levels of the unannotated RNAs predicted by Rockhopper based on the transcripts per million (TPM) [37]. A generalized linear model approach influenced by the multi-factorial EdgeR [38] was used to carry out a differential expression analysis. Genes that were expressed differentially were filtered according to previous stablished parameters [39,40] and considering a fold change ≥ 2 or ≤-2 and a FDR p-value ≤ 0.05. The putative sRNAs were classified as intergenic (encoded by intergenic regions) or antisense (encoded in the reverse direction of the putative ORFs). Only sRNAs differentially expressed when compared ammonia growth conditions to cyanide-dependent growth (sodium cyanide or jewelry wastewater) were curated manually and considered for further analyses. Thus, the genetic environment of each sRNA was analyzed manually and the nearest Rho-independent terminator to each sRNA was identified using the FindTerm [41] and ARNold [42] software. Bacterial promoters were predicted by using Prokaryotic Promoter Predictor [43] and Neutral Network Promoter Prediction (BDGP) [44] software. The presence of predicted promoter regions and Rho-independent transcription terminators was applied as selecting criteria. Mfold was used to predict sRNA secondary structures [45]. Potential sRNAs targets were identified by using the TargetRNA2 software [46]. To identify bacterial sRNA homologues, the GLASSgo software, the bacterial sRNA Database (BSRD) and the BLASTn were used [47,48].

### Identification of sRNAs by RT-PCR

Predicted sRNAs were amplified by RT-PCR using oligonucleotides specific for each gene (S1 Table). RNAs were isolated from *P*. *pseudoalcaligenes* CECT5344 cells cultured with ammonium chloride or cyanide from the jewelry residue (2 mM concentration in the media). The concentration and purity of the RNA samples were measured by using a ND1000 spectrophotometer (Agilent Technologies). Synthesis of total cDNA was achieved in 20 μl final volume containing 500 ng RNA, 0.1 mM dNTPs, 200 U SuperScript II Reverse Transcriptase (Invitrogen, California-USA), and 3.75 mM random hexamers (Applied Biosystems). Samples were initially heated at 65°C for 5 min and then incubated at 42°C for 50 min, followed by incubation at 70°C for 15 min. The cDNA was purified using Favorprep Gel/PCR purification kit from Favorgen Biotech Corporation (Taiwan) and the concentration was measured using a Nanodrop. PCR reactions were achieved in a 25 μl reaction (final volume), containing 2 μl of diluted cDNA (5 ng), 0.2 μM of each primer and 0.25μl of BioTaq DNA polymerase (Bioline Technologies). Samples were initially denatured by heating at 95°C for 3 min, followed by 40 cycles of amplification (95°C, 30 s; annealing temperature, 60°C, 30 s; elongation and signal acquisition, 72°C, 30 s). Data were normalized by using the *rpoB* gene as housekeeping. RT-PCR reactions were carried out with isolated RNAs from three independent biological replicates for each condition with ammonium or the cyanide-containing jewelry wastewater as the sole nitrogen source. PCR products were separated by electrophoresis using 3% agarose gels.

### Validation of sRNA targets by qRT-PCR

Putative gene targets of sRNAs were analyzed by qRT-PCR for each specific gene. RNAs were isolated from *P*. *pseudoalcaligenes* CECT5344 cells grown with ammonium chloride or cyanide from the jewelry residue and cDNA synthesis, purification and quantification were performed as described above. The iQ5 Multicolour Real-Time PCR Detection System (Bio-Rad) was used in a 25 μl reaction (final volume), containing 2 μl of diluted cDNA (12.5, 2.5 and 0.5 ng), 0.2 μM of each specific primer (S1 Table) and 12.5 μl of iQ SYBR Green Supermix (Bio-Rad). Target cDNAs and reference samples were amplified three times in separate PCR reactions. Samples were initially denatured by heating at 95°C for 3 min, followed by 40 cycles of amplification (95°C, 30 s; test annealing temperature, 60°C, 30 s; elongation and signal acquisition, 72°C, 30 s). For relative quantification of the fluorescence values, a calibration curve was made using dilution series from 80 to 0.008 ng of *P*. *pseudoalcaligenes* CECT5344 genomic DNA sample. Data were normalized by using the *rpoB* gene as housekeeping.

## Results

### Identification of putative sRNAs under cyanotrophic conditions in *P*. *pseudoalcaligenes* CECT5344

RNA-Seq was carried out from cells grown with a jewelry wastewater (containing 2 mM free cyanide), with 2 mM sodium cyanide or with 2 mM ammonium chloride as the sole nitrogen source. A differential analysis was developed initially when compared data from the jewelry wastewater *versus* ammonium, revealing the existence of 20 putative sRNAs ranging from 241 to 14 bp size (Table 1). A total of 16 sRNAs were classified as intergenic, while only 4 sRNAs were antisense. The sRNA expression fold changes were calculated considering the jewelry cyanide-containing media as the reference condition. From these 20 differentially expressed sRNAs, 13 were induced by the cyanide-containing residue, whereas 7 were repressed under cyanotrophic conditions. Most putative sRNAs identified in the strain CECT5344 were homologous to sRNAs previously described for other bacterial strains, except sRNA676 and sRNA679 that were exclusives of the strain CECT5344 (Table 1). A synthetic media with sodium cyanide was used for another differential analysis comparing data from sodium cyanide *versus* ammonium to elucidate sRNAs that were specifically up- or down-regulated by cyanide. A total of 15 sRNAs were shared between both comparative analyses (jewelry wastewater *versus* ammonium and sodium cyanide *versus* ammonium) while 5 sRNAs were specifically regulated by the residue (Table 1). Thus, specific sRNAs overexpressed in the jewelry residue (Table 1) were RNA184 (fold change 3.58), RNA559 (fold change 3.04), and RNA679 (fold change 2.13), whereas sRNAs specifically repressed in the jewelry wastewater were RNA150 (fold change -3.56) and RNA511 (fold change -2.06).

**Table 1:** Identification and characteristics of putative sRNAs involved in the detoxification of cyanide-containing wastewaters.

sRNA name	Size (pb)	Genome location (Start-Stop)	Frame	Type	Fold change (Res. vs NH4)	p-value (Res. vs NH4)	Fold change (CN vs NH4)	p-value (CN vs NH4)	Annotated previously	Identified RT-PCR
RNA14	38	123092–123130	-	Intergenic	-2.92	1.4E-06	-3.07	5.6E-07	Yes	Yes
RNA77	28	430235–430263	+	Intergenic	2.77	1.8E-07	2.50	2.9E-06	Yes	No
RNA149	31	852589–852620	+	Antisense	-4.68	1.4E-12	-2.38	4.9E-05	Yes	Yes
RNA150	241	852772–853013	+	Antisense	-3.76	5.8E-10	-	-	Yes	Yes
RNA184	45	1032809–1032854	-	Intergenic	3.58	4.8E-08	-	-	Yes	Yes
RNA222	51	1365111–1365162	+	Intergenic	2.08	4.3E-03	2.59	1.9E-04	Yes	Yes
RNA258	86	1664608–1664694	+	Intergenic	7.99	> 1.0E-17	7.54	> 1.0E-17	Yes	Yes
RNA312	40	2018669–2018709	+	Antisense	41.56	> 1.0E-17	22.64	> 1.0E-17	Yes	Yes
RNA431	37	2814997–2815034	-	Intergenic	2.15	6.4E-05	5.22	> 1.0E-17	Yes	Yes
RNA455	29	2995436–2995465	+	Intergenic	3.53	2.2E-16	4.16	> 1.0E-17	Yes	Yes
RNA502	34	3288590–3288624	-	Intergenic	-2.29	1.7E-2	-3.32	1.9E-03	Yes	No
RNA511	38	3405640–3405678	-	Intergenic	-2.06	7.3E-4	-	-	Yes	Yes
RNA559	38	3685225–3685263	+	Intergenic	3.04	2.1E-2	-	-	Yes	Yes
RNA601	124	4006653–4006777	-	Intergenic	-2.71	1.1E-09	-2.35	1.6E-07	Yes	Yes
RNA649	21	4241523–4241544	-	Antisense	2.59	6.5E-07	2.94	1.9E-08	Yes	Yes
RNA655	57	4337874–4337931	-	Intergenic	3.99	1.7E-13	6.69	> 1.0E-17	Yes	Yes
RNA675	22	4439338–4439360	+	Intergenic	3.40	2.4E-12	3.11	1.1E-10	Yes	No
RNA676	24	4440233–4440257	+	Intergenic	2.93	3.7E-08	2.27	3.2E-05	No	No
RNA679	79	4450665–4450744	-	Intergenic	2.13	5.0E-05	-	-	No	Yes
RNA683	14	4541161–4541175	+	Intergenic	-10.11	> 1.0E-17	-10.22	> 1.0E-17	Yes	Yes

*Appl Microbiol Biotechnol* 2008, **80**:42Seffernick JL, Samanta SK, Louie TM, Wackett LP, Subramanian M: **Investigative mining of sequence data for novel enzymes: A case study with nitrilases**. *J Biotech* 2009, **143**:17–26.

### Analysis of small RNAs putatively regulating cyanide wastewaters detoxification in *P*. *pseudoalcaligenes* CECT5344

**Fig 1:**
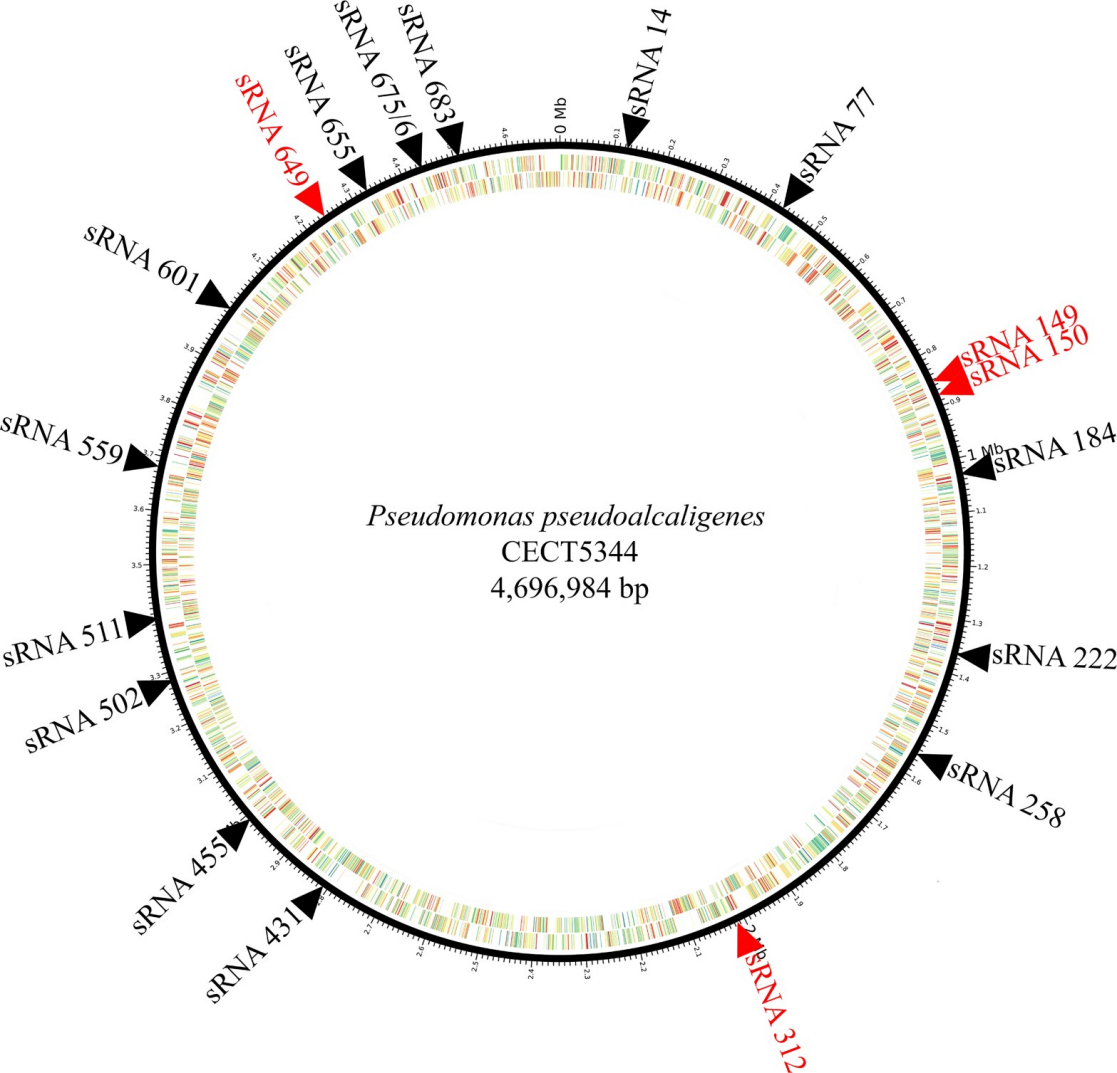
Localization of putative sRNAs differentially expressed (cyanide-containing wastewater *versus* ammonium) in the genome of *P*. *pseudoalcaligenes* CECT5344. The twenty sRNAs with differential expression (cyanide-containing wastewater/ammonium) are located within the genome of the strain CECT5344 (for genome information see reference [22]). The intergenic sRNAs are shown in black and the antisense sRNAs in red.

Localization of the 20 putative sRNAs in the whole genome sequence of *P*. *pseudoalcaligenes* CECT5344 was possible according to the previous information of the complete genome of this bacterial strain [20,21,22]. Most of the sRNAs were found very disperse in the genome of the strain CECT5344, except sRNA149-sRNA150 and sRNA675-sRNA683, which were closely located (Fig 1).

**Fig 2:**
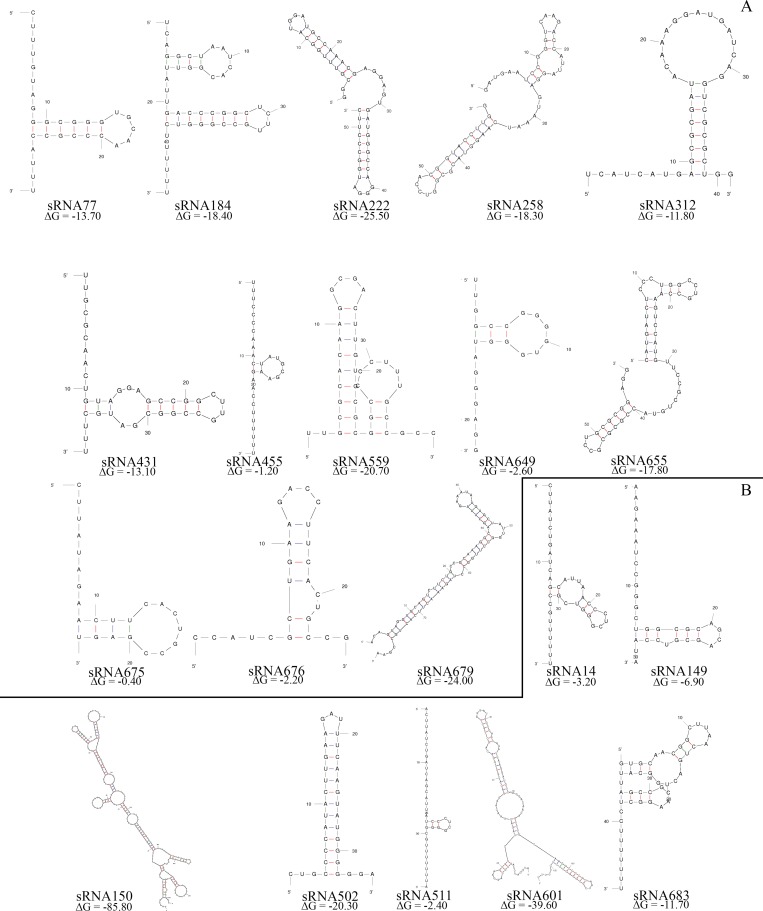
Prediction of the secondary structure of the 20 sRNAs with differential expression in *P*. *pseudoalcaligenes* CECT5344. The secondary putative structures of the identified sRNAs were predicted using the Mfold software [46]. **A.** Small RNAs induced by the jewelry wastewater. **B.** Small RNAs repressed by the jewelry wastewater.

The secondary structure of the 20 sRNAs identified was also predicted (Fig 2). The core algorithm has in consideration a minimum value free energy of Gibbs (ΔG) < 0 for the whole structure as well as for the folding of each base pair. Most of the 20 sRNAs were predicted to form highly structured molecules including more than one hairpin loop.

**Fig 3:**
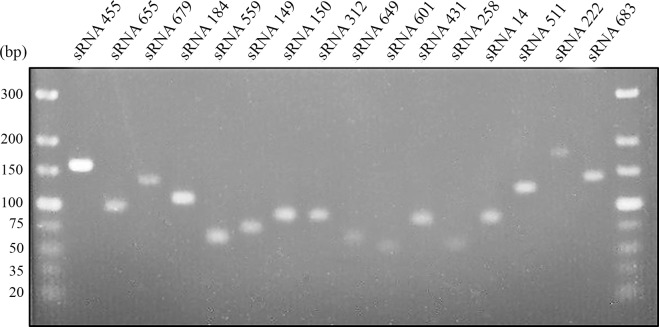
Detection by RT-PCR of the 20 sRNAs of *P*. *pseudoalcaligenes* CECT5344 with differential expression. Electrophoresis on 3% agarose gel of the PCR products corresponding to the 20 sRNAs induced or repressed in the jewelry wastewater. First and last lanes, TracKit Ultra Low Range DNA ladder (Invitrogen).

To confirm the existence of the 20 small RNAs identified by RNA-Seq that change their expression in the jewelry wastewater, a different methodology was applied based on RT-PCR (Fig 3). Of the 20 predicted sRNAs analyzed, 16 (80%) were positively amplified using specific oligonucleotides (S1 Table). These PCR products were further subjected to Sanger sequencing for their validation. Only these 16 sRNAs amplified by RT-PCR (Fig 3 and Table 1) were considered for further analyses.

### Bacterial distribution of *P*. *pseudoalcaligenes* CECT5344 sRNA candidates

**Fig 4:**
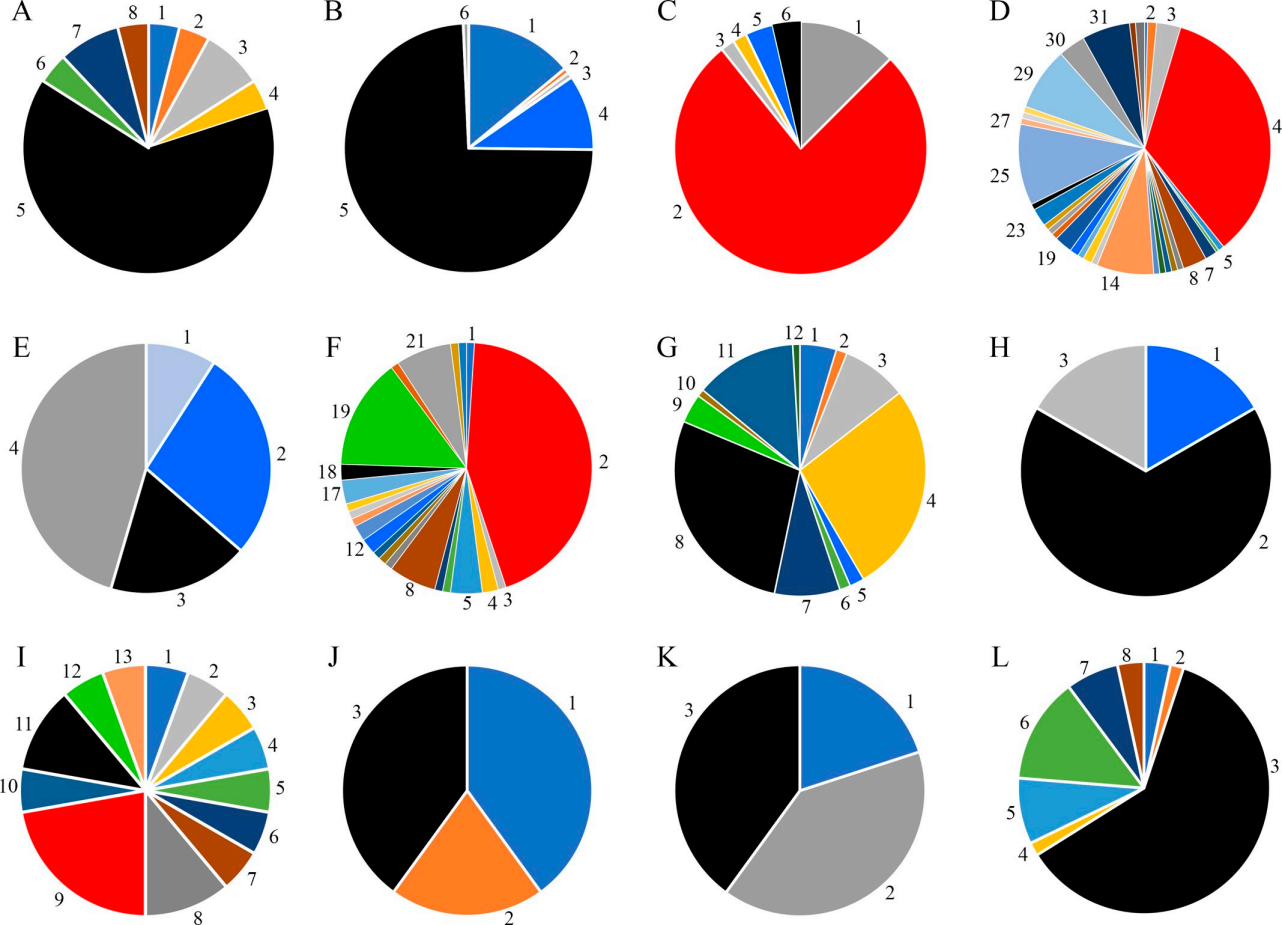
Distribution of *P. pseudoalcaligenes* CECT5344 sRNAs differentially expressed among bacteria. The bacterial sRNAs were analyzed using the GLASSgo software. 100% represents the total sRNAs sequences found in all bacterial species, considering the number of sequences as well as the % of identity (with at least 60% identity among sequences). Only sRNAs with homologs in more than two species are shown. The specific contribution (%) of each specie is indicated when it is higher than > 1%. **A.** (sRNA14): 1, *E*. *coli* (4.0); 2, *Oceanimonas sp*. (4.0); 3, *P*. *chlororaphis* (8.0); 4, *P*. *plecoglossicida* (4.0); 5, *P*. *pseudoalcaligenes* (64.0); 6, *Shigella dysenteriae* (4.0); 7, *Shigella sonnei* (8.0); 8, *Yersinia enterocolitica* (4.0). **B.** (sRNA149): 1, *P*. *stutzeri* (12.5); 2, *P*. *aeruginosa* (76.8); 3, *P*. *fulva* (1.8); 4, *P*. *mandelii* (1.8); 5, *P*. *mendocina* (3.6); 6, *P*. *pseudoalcaligenes* (3.6). **C.** (sRNA184): 2, *Azotobacter vinelandii* (1.2); 3, *Pseudomonadaceae bacterium* (3.1); 4, *P*. *aeruginosa* (34.6); 7, *P*. *brassicacearum* (1.5); 8, *P*. *chlororaphis* (3.1); 14, *P*. *fluorescens* (7.3); 16, *P*. *knackmussii* (1.2); 18, *P*. *mendocina* (1.2); 19, *P*. *monteilii* (2.3); 23, *P*. *protegens* (2.3); 25, *P*. *putida* (10.4); 29, *Pseudomonas* sp. (8.1); 30, *P*. *stutzeri* (3.5); 31, *P*. *syringae* (6.2); 33, uncultured soil microorganism (1.2); **D.** (sRNA222): 1, *P*. *fulva* (9.1); 2, *P*. *mendocina* (27.3); 3, *P*. *pseudoalcaligenes* (18.2); 4, *P*. *stutzeri* (45.5). **E.** (sRNA258): 2, *P*. *aeruginosa* (43.9); 4, *P*. *brassicacearum* (2); 5, *P*. *chlororaphis* (4.1); 8, *P*. *fluorescens* (6.1); 12, *P*. *mendocina* (2); 13, *P*. *monteilii* (2); 17, *P*. *protegens* (3.1); 18, *P*. *pseudoalcaligenes* (2); 19, *P*. *putida* (14.3); 21, *Pseudomonas*. sp. (7.1). **F.** (sRNA431). 1, *P*. *chlororaphis* (4.7); 2, *P*. *deceptionensis* (1.4); 3, *P*. *entomophila* (8.4); 4, *P*. *fluorescens* (27.1); 5, *P*. *mendocina* (1.9); 6, *P*. *mosselii* (1.4); 7, *P*. *protegens* (8.4); 8, *P*. *pseudoalcaligenes* (28); 9, *P*. *putida* (3.7); 11, *Pseudomonas*. sp. (13.1). **G.** (sRNA455): 1, *P*. *mendocina* (16.7); 2, *P*. *pseudoalcaligenes* (66.7), 3, *Helicobacter cetorum* (16.7). **H.** (sRNA559): 1, *Alcanivorax dieselolei* (5.5); 2, *Comamonas testosteroni* (5.5); 3, *Cronobacter muytjensii* (5.5); 4, *Cronobacter universalis* (5.5); 5, *Enterobacter cloacae* (5.5); 6, *Klebsiella oxytoca* (5.5); 7, *Methylophaga frappieri* (5.5); 8, *Pantoea sp*. (11.1); 9, *P*. *aeruginosa* (22.2); 10, *P*. *fluorescens* (5.5); 11, *P*. *pseudoalcaligenes* (11.1); 12, *P*. *putida* (5.5); 13, *Pseudomonas* sp. (5.5); **I.** (sRNA601): 1, *P*. *mendocina* (40); 2, *P*. *fulva* (20); 3, *P*. *pseudoalcaligenes* (40). **J.** (sRNA649): 1, *P*. *extremaustralis* (20); 2, *P*. *stutzeri* (40); 3, *P*. *pseudoalcaligenes* (40). **K.** (sRNA683): 1, *E*. *coli* (3.4); 2, *Oceanimonas* sp. (1.7); 3, *P*. *pseudoalcaligenes* (61); 4. *Rahnella aquatilis* (1.7); 5, *Shigella boydii* (8.5); 6. *Shigella flexneri* (13.6); 7, *Shigella sonnei* (6.8); 8, *Vibrio vulnificus* (3.4).

From the 16 selected sRNAs, 11 sRNAs were conserved in more than two bacterial strains (Fig 4), whereas sRNA150, sRNA312, sRNA511 and sRNA655 were poorly distributed (not shown). In addition, as mentioned above, sRNA679 did not show homologues among bacteria and should be considered exclusives of the strain CECT5344 (Table 1).

Most of the *P*. *pseudoalcaligenes* CECT5344 sRNA candidates were found in γ-proteobacteria, specifically in pseudomonads. sRNA14 showed a high representation among enterobacteria (Fig 4A). sRNA149 and sRNA222 were exclusively found in *Pseudomonas* (Fig 4B and 4D). Widely distributed among bacterial genera were sRNA184, sRNA258, sRNA431 and sRNA559 (Fig 4C, 4E, 4F and 4H). sRNA184 was highly represented in *Pseudomonas* but was also found in *Azotobacter* (Fig 4C), whereas sRNA431 was present in *Rhodanobacter*, which belongs to the Xanthomonadaceae family (Fig 4F). sRNA455 was poorly distributed, being represented in two *Pseudomonas* strains and in *Helicobacter*, an ε-proteobacteria (Fig 4G). By contrast, sRNA559 was the most widely distributed among bacteria, including the β-proteobacteria from the Comamonadaceae family and γ-proteobacteria strains belonging to Alcanivoraceae, Piscirickettsiaceae and Pseudomonadaceae families (Fig 4H). sRNAs 601 (Fig 4I) and sRNA649 (Fig 4J) were found mainly in *Pseudomonas*, whereas sRNA683 (Fig 4K) was mainly present in the Enterobacteriaceae family.

### Identification of predicted targets for *P*. *pseudoalcaligenes* CECT5344 sRNAs expressed under cyanotrophic conditions

**Fig 5:**
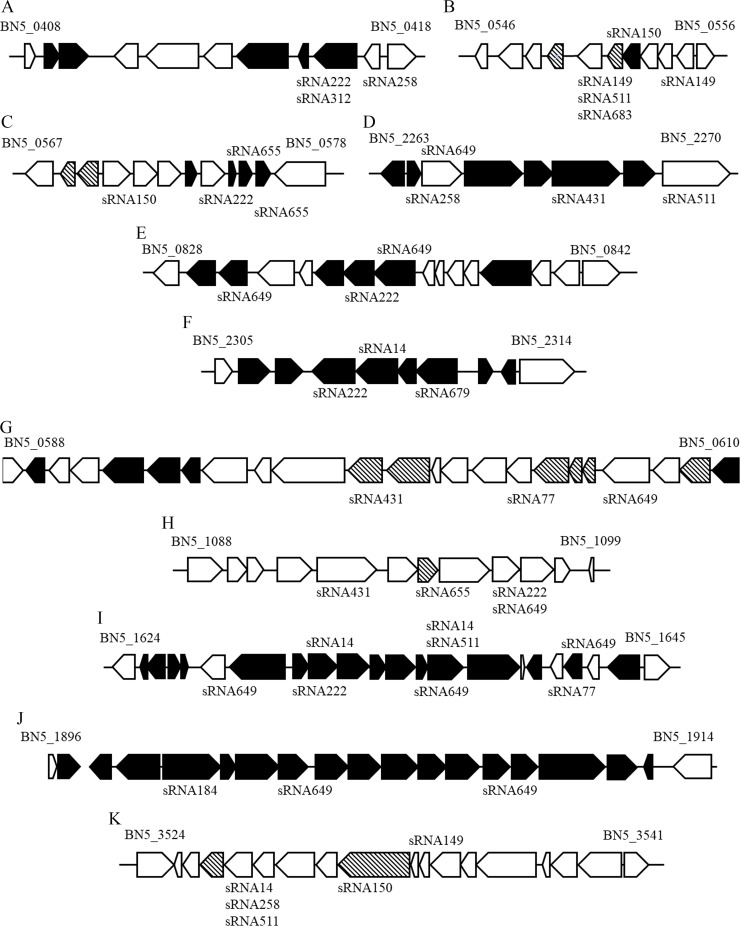
*P*. *pseudoalcaligenes* CECT5344 gene clusters putatively regulated by small RNAs in response to cyanide-containing wastewaters. Black genes, induced by the jewelry wastewater; dashed genes, repressed by the jewelry wastewater, as previously described in other omic studies [27,28]. These gene clusters (with gene references corresponding to HG916826 accession number [25]) code for the following putative proteins: **A.** BN5_0408, polyhydroxyalkanoic acid system protein; BN5_0409 and BN5_0410, poly(hydroxyalkanoate) granule-associated proteins; BN5_0411, TetR family transcriptional regulator phaD; BN5_0412, polyhydroxyalkanoate synthase, class II; BN5_0413, poly(3-hydroxyalkanoate) depolymerase; BN5_0414, poly(3-hydroxyalkanoate) polymerase; BN5_0415, putative phosphoribosylformimino-5-aminoimidazole carboxamide riboside isomerase; BN5_0416, ATP-dependent protease ATPase subunit HslU; BN5_0417, ATP-dependent protease subunit HslV; BN5_0418, uncharacterized protein. **B.** BN5_0546, azurin; BN5_0547, uncharacterized protein; BN5_0548, cytokinin riboside 5'-monophosphate phosphoribohydrolase; BN5_0549, lysine exporter protein LysE/YggA; BN5_0550, glycerophosphodiester phosphodiesterase; BN5_0551, hydrogenase/urease protein; BN5_0552, urease accessory protein UreG; BN5_0553, urease protein UreF; BN5_0554, urease accessory protein UreE; BN5_0555, TetR family transcriptional regulator; BN5_0556, ferredoxin. **C.** BN5_0567 and BN5_0568, membrane proteins; BN5_0569, uncharacterized protein; BN5_0570, periplasmic binding protein/LacI transcriptional regulator; BN5_0571 and BN5_0572, extracellular solute-binding proteins; BN5_0573, type II secretion system protein; BN5_0574, oxaloacetate decarboxylase; BN5_0575, uncharacterized protein; BN5_0576, hemerythrin HHE cation binding domain-containing protein; BN5_0577, uncharacterized protein; BN5_0578, urease subunit alpha-urea amidohydrolase subunit alpha. **D.** BN5_2263, putative short-chain dehydrogenase; BN5_2264, MerR family transcriptional regulator; BN5_2265, isovaleryl-CoA dehydrogenase; BN5_2266, propionyl-CoA carboxylase, beta subunit; BN5_2267, γ-carboxygeranoyl-CoA hydratase; BN5_2268, 3-methylcrotonoyl-CoA carboxylase, alpha subunit; BN5_2269, hydroxymethylglutaryl-CoA lyase; BN5_2270, acetoacetyl-CoA synthetase. **E.** BN5_0828, adenine deaminase/aminohydrolase; BN5_0829, oxidoreductase-2OG-Fe(II) oxygenase family; BN5_0830, basic membrane lipoprotein; BN5_0831, hydroxydechloroatrazine ethylaminohydrolase; BN5_0832, uncharacterized protein; BN5_0833, ABC transporter- permease protein; BN5_0834, inner membrane translocator; BN5_0835, ABC transporter-ATP-binding protein; BN5_0836, short chain dehydrogenase; BN5_0837-BN5_0839, uncharacterized proteins; BN5_0840, TetR family transcriptional regulator; BN5_0841, LysR family transcriptional regulator; BN5_0842, β-alanine-pyruvate transaminase; **F.** BN5_2305, transposase for insertion sequence element IS1106; BN5_2306, xanthine dehydrogenase accesory protein; BN5_2307, AraC-family regulatory protein; BN5_2308, aminotransferase; BN5_2309, glutamine synthetase; BN5_2310, XRE family transcriptional regulator; BN5_2311, FAD-dependent oxidoreductase; BN5_2312, phage integrase; BN5_2313, CopG family transcriptional regulator; BN5_2314, transposase for insertion sequences IS1326/IS1353. **G.** BN5_0588, α-adducin; BN5_0589 and BN5_0590, ABC transporter proteins; BN5_0591 and BN5_0592, inner-membrane translocator proteins; BN5_0593, ABC-type branched-chain amino acid transport system-periplasmic component protein; BN5_0594, GntR family transcriptional regulator; BN5_0595, allophanate hydrolase; BN5_0596, UPF0056 membrane protein; BN5_0597, integral membrane sensor hybrid histidine kinase; BN5_0598, phosphoribosylamine-glycine ligase-glycinamide ribonucleotide (GARS)/phosphoribosylglycinamide synthetase; BN5_0599, bifunctional purine biosynthesis protein PurH (IMP cyclohydrolase-IMP synthase and phosphoribosylaminoimidazolecarboxamide formyltransferase (AICAR transformylase); BN5_0600, Fis family DNA-binding protein; BN5_0601, tRNA-dihydrouridine synthase; BN5_0602, zinc finger-domain-containing protein; BN5_0603, ribosomal protein L11 methyltransferase BN5_0604, acetyl-CoA carboxylase biotin carboxylase subunit; BN5_0605, acetyl-CoA carboxylase biotin carboxyl carrier protein subunit; BN5_0606; 3-dehydroquinate dehydratase-3-dehydroquinase type II; BN5_0607, thiol:disulfide interchange protein DsbD-protein-disulfide reductase; BN5_0608, methyl-accepting chemotaxis protein; BN5_0609, response regulator receiver modulated diguanylate cyclase; BN5_0610, uncharacterized protein. **H.** BN5_1088, oxidoreductase GMC family; BN5_1089, autotransporter β-domain protein; BN5_1090, extracellular serine protease; BN5_1091, peptide chain release factor 2; BN5_1092, lysine-tRNA ligase LysRS; BN5_1093, HTH-type transcriptional regulator BetI; BN5_1094, lipoprotein; BN5_1095 and BN5_1096, uncharacterized proteins; BN5_1097, lipoprotein; BN5_1098 and BN5_1099, uncharacterized proteins. **I.** BN5_1624, ATPase; BN5_1625 and BN5_1626, uncharacterized proteins; BN5_1627 and BN5_1628, cysteine synthase; BN5_1629, inner membrane transporter YiJE; BN5_1630, σ^54^-dependent transcriptional regulator NitA; BN5_1631, uncharacterized protein NitB; BN5_1632, nitrilase NitC; BN5_1633, radical SAM domain-containing protein-biotin synthase enzyme-ribosomal RNA large subunit methyltransferase N NitD; BN5_1634, acetyltransferase-GCN5-related N-acetyltransferase NitE; BN5_1635, AIR synthase related protein domain protein NitF; BN5_1636, uncharacterized protein NitG; BN5_1637, FAD-dependent oxidoreductase NitH; BN5_1638, isocitrate dehydrogenase kinase/phosphatase-IDH; BN5_1639, uncharacterized protein; BN5_1640, β-lactamase domain-containing protein; BN5_1641, inner membrane protein; BN5_1642, YecA family protein; BN5_1643, RNA-directed DNA polymerase; BN5_1644, uncharacterized protein; BN5_1645, RNA-directed DNA polymerase. **J.** BN5_1896, uncharacterized protein; BN5_1897, thiol-disulfide isomerase and thioredoxins protein; BN5_1898, 2-dehydro-3-deoxyphosphogluconate/4-hydroxy-2-oxoglutarate aldolase; BN5_1899, GntR family transcriptional regulator; BN5_1900, sulfite reductase-NADPH hemoprotein β-component; BN5_1901, uncharacterized protein; BN5_1902, terminal oxidase subunit I CioA; BN5_1903, cytochrome *d* ubiquinol oxidase, subunit II CioB; BN5_1904, phosphoserine/phosphohydroxythreonine aminotransferase; BN5_1905, histidinol phosphate/imidazole acetol phosphate transaminase; BN5_1906, acetylornithine aminotransferase; BN5_1907, 4-hydroxy-tetrahydrodipicolinate synthase-HTPA synthase; BN5_1908, high-affinity glucose transporter; BN5_1909, methylenetetrahydrofolate reductase; BN5_1910, cysteine synthase; BN5_1911, NADP-dependent malic enzyme; BN5_1912, nitrilase; BN5_1913, uncharacterized protein; BN5_1914, putative RuBisCO transcriptional regulator. **K.** BN5_3524, transcriptional regulator XRE family; BN5_3525, uncharacterized protein; BN5_3526, conjugation TrbI family protein; BN5_3527, conjugal transfer protein TrbG/VirB9/CagX; BN5_3528, conjugal transfer protein TrbF; BN5_3529, conjugal transfer protein TrbL/VirB6; BN5_3530, conjugal transfer protein TrbJ; BN5_3531, P-type conjugative transfer protein TrbE; BN5_3532, conjugal transfer transmembrane protein TrbD; BN5_3533, conjugal transfer transmembrane protein TrbC; BN5_3534, Type II secretion system protein E; BN5_3535, uncharacterized protein; BN5_3536, TRAG family protein; BN5_3537, uncharacterized protein; BN5_3538, LysR family transcriptional regulator; BN5_3539, major facilitator superfamily protein; BN5_3540, helix-turn-helix domain protein; BN5_3541, uncharacterized protein.

The 16 sRNAs candidates were predicted to have a wide range of targets (S2 Table), according to TargetRNA2 software [46]. Furthermore, an elevated number of *P*. *pseudoalcaligenes* CECT5344 target genes were predicted to be arranged in gene clusters or located in the same *locus* (Fig 5). Thus, the small transcripts sRNA222, sRNA312 and sRNA258, which were found upregulated in the jewelry residue, were predicted to regulate (at transcriptional, translational or post-translational level) components involved in the metabolism of medium-length chain polyhydroxyalkanoates (Fig 5A). Also, sRNA222 and sRNA312, share as target the *hisA* gene (BN5_0415) that encodes a putative phosphoribosylformimino-5-amidazole carboxamide ribotide isomerase involved in the synthesis of histidine and inositol monophosphate. The sRNA149, sRNA511 and sRNA683 (Fig 5B) were downregulated by the jewelry residue and were predicted to regulate components involved in the synthesis of phospholipids (BN5_0550) and urea metabolism (BN5_0551 and BN5_0552). Other sRNAs putatively involved in controlling components related with lipids metabolism were sRNA258, sRNA649 and sRNA431, which were upregulated by the jewelry residue, and sRNA511 (Fig 5D). The sRNA150, sRNA222, and sRNA655 were predicted to modulate components related to an oxaloacetate decarboxylase encoded by the BN5_0574 gene and an hemerythrin-like protein encoded by the BN5_0576 gene (Fig 5C). The sRNA222 and sRNA649 displayed different targets belonging to the BN5_0828-BN5_0842, including an inner membrane translocator efflux pump (BN5_0834/W6QTZ9) (Fig 5E). A gene cluster (BN5_2305-BN5_2314) highly targeted by the sRNAs codes for a putative xanthine dehydrogenase accessory protein, an aminotransferase (targeted by sRNA222), the glutamine synthetase (regulated by sRNA14), an FAD-dependent oxidoreductase (targeted by sRNA679) and a CopG-family transcriptional regulator (Fig 5F). Highly targeted by sRNA649, and in a lower extent by sRNA14, sRNA222, sRNA551 and sRNA184, were two gene clusters previously described to play a key role on cyanide metabolism in the strain CECT5344; the *nit1C* gene cluster (BN5_1630-BN5_1637) encoding the nitrilase NitC essential for cyanide assimilation (Fig 5I) and the *cio* gene cluster (BN5_1899-BN5_1913) that codes for the cyanide insensitive terminal oxidase CioAB (Fig 5J). Thus, four genes belonging to the *nit1C* gene cluster were predicted to be modulated by sRNAs: the *nitB* gene (BN5_1631/H9N5E2) was predicted to be controlled by sRNA222, the *nitC* gene (BN5_1632/H9N5E1) was putatively modulated by sRNA14, the *nitH* gene (BN5_1637) seemed to be targeted by sRNA14, sRNA511 and sRNA683, and the *nitG* gene (BN5_1636/H9N5D9) may be modulated by sRNA649 (Fig 5I). Also, components encoded by the *cio* gene cluster that were putatively regulated by sRNAs were an NADPH-dependent sulfite/nitrite reductase subunit (BN5_1900/W6RF17) that may be targeted by sRNA184 and the alternative oxidase *cioB* (BN5_1903/W6QVH5) and the methylenetetrahydrofolate reductase (BN5_1909/W6QWY1) that could be controlled by sRNA649 (Fig 5J). The sRNA14, sRNA258, sRNA511, sRNA150 and sRNA149 could regulate components related with conjugational DNA transfer processes like the conjugal transfer TrbD protein encoded by the BN5_3532 gene (Fig 5K).

Interestingly, some non-clustered targets of the 16 identified sRNAs by RT-PCR that were found up- or down-regulated in response to the jewelry wastewater in previous omic studies [23,24], included the arsenate reductase (BN5_1993/W6QVQ7) as target of sRNA14, an antioxidant AhpC/Tsa family member (BN5_4165) as target of sRNA150, and the methionine import ATP-binding protein MetN (BN5_4324/W6R3V7) as target of sRNA184 (S2 Table). The small RNA222 showed a high number of specific targets induced by the jewelry residue like a formate dehydrogenase (BN5_0294/W6QSE5), among others. The sRNA258 was predicted to regulate the wastewater-induced putative tricarboxylic transport membrane component (BN5_2918/W6QZP0) and a 4-oxalocrotonate decarboxylase (BN5_3617/W6RK34). Targets of sRNA312 induced by the jewelry residue were a formate dehydrogenase (BN5_0294/W6QSE5) and a putative anthranilate phosphoribosyl transferase (BN5_1879/W6QU73). A formamidase-formamide amidohydrolase (BN5_3204/W6QY63) was a predicted target of sRNA655 (S2 Table). sRNA649 showed the highest number of targets induced in the cyanide-containing wastewater, including a dihydropyrimidinase (BN5_0330/W6RAR6), the ferric uptake regulation (Fur) protein (BN5_0907/W6QZB3) and a regulatory GntR-like family member (BN5_1352/W6QVD8). By contrast, sRNA150, sRNA455, sRNAA511, sRNA559 and sRNA679 showed a very reduced number of specific targets that were found affected by the jewelry residue in previous omic studies in the strain CECT53444 (S2 Table).

Additionally, several targets of the 16 identified sRNAs were not described as cyanide-regulated genes/proteins in previous omic studies, such as a furoyl-CoA dehydrogenase (BN5_2298/W6RG72) targeted by sRNA184, the oxaloacetate dehydrogenase (BN5_0574/W6QYF7) and an *S*-adenosylhomocysteine deaminase (BN5_1672/W6QWB1) targeted by sRNA222, a homoserine *O*-acetyltransferase MetX (BN5_3973/W6R090) targeted by sRNA258 and sRNA511, an ornithine utilization regulator (BN5_2771/W6R4P0) as target of sRNA258, a siderophore biosynthesis component (BN5_1295/W6R0F2) as target of sRNA312 and sRNA649, the periplasmic nitrate reductase maturation protein NapD (BN5_2778/W6QZA5), an arsenate reductase (BN5_2786/W6R4Q4), a TetR transcriptional regulator (BN5_3897/W6RKR8) targeted by sRNA312, a glycerophosphodiester phosphodiesterase (BN5_0550/W6QRN4) as target of sRNA511, sRNA149 and sRNA683, a putative efflux pump outer membrane component TtgC (BN5_0813/W6QSE0), an alkyl hydroperoxide reductase AhpD (BN5_4391/W6R1I6), and a putative mercury resistance protein MerE (BN5_4479/W6R4C0) as targets of sRNA 511, and aliphatic nitrilase (BN5_1925/W6RF39), the periplasmic nitrate reductase electron transfer component NapB (BN5_2780/W6QWV3), and a phosphopanteine adenylyl transferase (BN5_4000/W6R1F1) as predicted targets of sRNA601, an acetylornithine deacetylase (BN5_0273/W6QQU2), a citrate transporter (BN5_0450/W6RB28), an organic hydroperoxide resistant component (BN5_2297/W6QY00), an NADPH-dependent 7-cyano-7-deazaguanine reductase (BN5_2508/w6QX49), and a cobyrinic acid ac-diamide synthase (BN5_3545/W6R060) as targets of sRNA649, and an ArsR family transcriptional regulator (BN5_0341/W6QPT4) as putative target of RNA679 (S2 Table).

To gain a direct evidence that the putative gene targets (Fig 5) are regulated by the identified sRNAs, a quantitative RT-PCR was carried out to obtain the gene expression rate from cells grown in the jewelry residue *versus* ammonium as nitrogen source (Table 2). All these gene targeted by sRNAs were significantly induced by the jewelry residue, especially those encoded by the *nit1C* gene cluster (BN5_1631-BN5_1637) and the *cioAB locus* (BN5_1900-BN5_1909).

**Table 2:** Quantitative RT-PCR of sRNAs target genes.

sRNAs	Gene targets	Ammonium (A)	Residue (R)	R/A ratio
222, 312	BN5_0415	7.4 ± 1.8	109.5 ± 9	14.8
258	BN5_0417	18.3 ± 1.2	348.4 ± 12	19.0
149, 511, 683	BN5_0550	0.9 ± 0.06	9.8 ± 0.8	10.9
150	BN5_0551	0.6 ± 0.04	38.8 ± 4.9	64.7
149	BN5_0554	0.5 ± 0.07	4.8 ± 0.9	9.6
150	BN5_0570	1.4 ± 0.1	6.1 ± 0.6	4.4
222	BN5_0574	5.1 ± 0.78	18.6 ± 0.3	3.6
655	BN5_0575	76.0 ± 5.2	226.9 ± 19	3.0
655	BN5_0577	0.3 ± 0.03	1.5 ± 0.02	5.0
431	BN5_0598	0.6 ± 0.05	5.3 ± 0.4	8.8
649	BN5_0607	9.5 ± 0.9	145.1 ± 10	15.3
649	BN5_0830	0.3 ± 0.02	2.5 ± 0.2	8.3
222	BN5_0834	0.3 ± 0.04	1.3 ± 0.1	4.3
649	BN5_0835	0.1 ± 0.03	1.1 ± 0.02	11.0
431	BN5_1092	9.3 ± 0.48	38.8 ± 4.0	4.2
655	BN5_1094	4.2 ± 0.05	17.4 ± 1.5	4.1
222, 649	BN5_1096	2.3 ± 0.2	12.9 ± 1.1	5.6
649	BN5_1629	0.8 ± 0.07	25.9 ± 1.8	32.4
222	BN5_1631	0.9 ± 0.08	1096 ± 76	1217
14	BN5_1632	0.8 ± 0.09	714 ± 31	893
649	BN5_1636	1.1 ± 0.1	1113 ± 84	1012
14, 511, 683	BN5_1637	0.7 ± 0.09	525 ± 48	750
649	BN5_1642	10.5 ± 0.7	27.7 ± 0.1	2.6
184	BN5_1900	5.7 ± 0.4	5059 ± 89	887
649	BN5_1903	5.5 ± 0.3	1877 ± 162	341
649	BN5_1909	1.7 ± 0.1	206.1 ± 18	153
258	BN5_2264	9.6 ± 1.9	186.7 ± 13	19.4
649	BN5_2265	1.2 ± 0.05	24.2 ± 1.4	20.2
431	BN5_2268	2.2 ± 0.08	39.9 ± 2.2	18.1
222	BN5_2308	12.3 ± 0.8	143.9 ± 2.3	11.7
14	BN5_2309	1.2 ± 0.07	50.4 ± 4.6	42.0
679	BN5_2311	2.1 ± 0.1	18.4 ± 1.9	8.8
14, 258, 511	BN5_3528	0.5 ± 0.05	17.9 ± 1.2	35.8
150	BN5_3532	0.2 ± 0.02	7.7 ± 0.4	38.5
149	BN5_3533	0.4 ± 0.04	16.7 ± 1.3	41.7

Expression of genes targeted by sRNAs was quantified using RNAm from cells grown with ammonium (A) or the jewelry residue (R) to determine the gene expression ratio (residue/ammonium). Gene targets are named according to their ID code [25].

## Discussion

Large amounts of cyanide-containing industrial wastes are generated worldwide and biological treatments to degrade cyanide have been demonstrated to be a powerful technology for cyanide removal [13]. The alkaliphilic bacterium *P*. *pseudoalcaligenes* CECT5344 degrades cyanide, metal–cyanide complexes and other cyano–derivatives [9,19]. Cyanide removal from jewelry wastewaters composed of metals like iron, copper and zinc, free cyanide and metal–cyanide complexes has been achieved in a reactor with *P*. *pseudoalcaligenes* CECT5344 [24]. The sequencing of the whole genome of the strain CECT5344 [20,21,22] has allowed to perform further omic studies in response to cyanide-containing wastewaters. Thus, DNA-microarrays has been generated and about 60 genes of *P*. *pseudoalcaligenes* CECT5344 have been described to be specifically induced by the jewelry residue [23]. In addition, a proteomic approach by quantitative LC-MS/MS was carried in response to the jewelry residue, yielding about 50 proteins specifically induced by this cyanide-containing waste [24], most of them encoded by genes found induced by cyanide in the DNA microarrays analysis. From these omic approaches, a high correlation was found between gene/protein induced, including metabolic enzymes, regulators and metal transporters, which have been described to have a key role for cyanide detoxification by *P*. *pseudoalcaligenes* CECT5344.

Bacterial small RNAs have evolved to modulate the expression of targeted genes in response to changes in the environment [31,49]. Different mechanisms of action for sRNAs have been described, including antisense sRNAs that act by base pairing to targeted mRNAs, and the sRNAs with limited complementarity with their targets, which usually required RNA chaperones like Hfq to activate/repress translation [28,29,30,31]. Also, there are sRNAs that regulate proteins, by binding and affecting protein activity, or by sequestering regulatory proteins, such as transcription factors [50]. It is worth nothing that *P*. *pseudoalcaligenes* CECT5344 presents in its genome a Hfq-encoding gene (BN5_0521) that could mediate sRNAs regulation.

To deeply understand the biodegradative process of cyanide-containing wastes by the strain CECT5344, an RNA-Seq analysis has been performed in response to the jewelry residue. Twenty putative sRNAs were found differentially expressed in the presence of the liquid cyanide-containing residue (Fig 1 and Table 1). The putative 20 sRNAs were predicted to form highly structured molecules including more than one hairpin loop (Fig 2). In cells grown with a synthetic media with sodium cyanide 15 of these 20 sRNAs were also identified (Table 1), suggesting that the remained five sRNAs (sRNA150, sRNA184, sRNA511, sRNA559 and sRNA679) may have a regulatory role in detoxification of metals and metalloids that are also present in the jewelry wastewaters. Many of these sRNAs have the potential to form complex conformations similarly to those commonly associated with many other directly acting RNA transcripts, including known bacterial sRNAs [50,51]. From the 20 sRNAs analyzed, 16 sRNAs were amplified by RT-PCR (Fig 3), a percentage of success (80%) higher than those described in other studies [51,52,53]. Antisense RNAs, which are transcribed from the DNA strand opposite to the genes they regulate and display complementarity to their target RNA, are often located in the untranslated regions (UTRs) of the corresponding gene. The RNA duplex formation can affect ribosome binding, translation, termination processes or mRNA stability by rearranging hairpins in the target RNA [54]. The sRNAs candidates to play a role in cyanide detoxification from jewelry wastewaters were subjected to phylogenetic analysis. Most *P*. *pseudoalcaligenes* CECT5344 sRNAs were found in γ-proteobacteria, specifically in pseudomonads (Fig 4). Considering that sRNA679 was exclusive of the cyanothrophic strain CECT5344, and that it displays 14 putative targets not identified in previous transcriptomic and proteomic approaches in this strain (S2 Table), it can be proposed that sRNA679 might regulate cyanide-degradation processes at a post-translational level, as proposed for most bacterial sRNAs [49]. The five wastewater-specific sRNAs identified in the differential analysis of jewelry residue *versus* sodium cyanide (sRNA150, sRNA184, sRNA511, sRNA559 and sRNA679) were exclusives of the strain CECT5344 or very poorly distributed among bacteria, highlighting their relevance in the control of detoxification of other hazards, apart from cyanide, present in this wastewater. In addition, the low distribution of sRNA222 and sRNA649 and the high number of their targets related with cyanide detoxification suggest that these sRNAs may play a role regulating the different processes involved in cyanide metabolism (Fig 5 and S2 Table). Thus, sRNA649 shows the highest number of targets (S2 Table) and has the major impact on the regulation of numerous genes that are arranged in gene clusters with an essential role in cyanide assimilation and resistance, such as the *nit1C* gene cluster (Fig 5I) that codes for the nitrilase NitC essential for cyanide assimilation [26] and the *cio* gene cluster (Fig 5J) that encodes the cyanide-insensitive alternative oxidase CioAB [25]. Also, sRNA222 has as targets the BN5_0415 gene located upstream a mcl-PHA polymerase involved in the metabolism of medium length polyhydroxyalkanoates (BN5_0415), an aminotransferase gene (BN5_2309) located downstream the glutamine synthetase gene, a formate dehydrogenase encoded by BN5_0294, and the *nitB* gene (BN5_1631) from the *nit1C* cluster. The specific role of NitB remains unknown, but as mentioned above, the *nit1C* gene cluster of *P*. *pseudoalcaligenes* CECT5344 is essential for cyanide detoxification [26]. The ability of the strain CECT5344 to accumulate polyhydroxyalkanoates during cyanide removal from jewelry wastewaters has been demonstrated previously, thus adding value to the degradation process by production of bioplastics [27]. The induction of the glutamine synthetase and other enzymes involved in nitrogen assimilation like urease could be related to a nitrogen starvation response considering cyanide toxicity and its low concentration in the media (2 mM). Also, putative targets of the sRNA14 were the nitrilase NitC (BN5_1632/H9N5E1), the FAD-dependent oxidoreductase NitH (BN5-1637/H9N5D8), an arsenate reductase gene (BN5_1993/W6QVQ7) and the glutamine synthetase (BN5_2309/W6QVI3). Although arsenate has not been found in the jewelry wastewater, the induction of arsenate reductase by this residue has been previously observed [23].

Interestingly, a formamidase-formamide amidohydrolase (BN5_3204/W6QY63) was described as predicted target of sRNA655. This enzyme produces formic acid, which could be converted into CO_2_ by the action of a formate dehydrogenase. These two enzymes have been described as key enzymes on several bacterial hydrolytic pathways for cyanide degradation [2]. Thus, cyanide assimilation/detoxification in the strain CECT5344 could not only occur through the nitrilase NitC, but also by the action of a formamidase/formate dehydrogenase that could have a complementary function in this process. This hypothesis could be supported considering that the formate dehydrogenase may be a component highly regulated in *P*. *pseudoalcaligenes* CECT5344, because it is also a target of sRNA222 and sRNA312. The formation of formamide occurs through the action of a cyanide hydratase, and several putative annotated nitrilases/cyanide hydratases were induced by the jewelry residue in the strain CECT5344 that could produce this intermediate in response to cyanide, as those encoded by the BN5_0736, BN5_3251 and BN5_4427 genes.

The conjugal transfer TrbD transmembrane protein (BN5_3532/ W6RJU5), which is repressed in the cells grown with the jewelry residue, is putatively regulated by sRNA150. The conjugation is a method widely used for bacterial DNA acquisition that has been previously described to be regulated by sRNA. Thus, in *Salmonella* the assembly of the conjugation machinery is strictly controlled by sRNAs under stress conditions [50], and it could to be also affected during cyanide detoxification in the strain CECT5344.

The wastewater-induced sRNA184 putatively causes the upregulation of an NADPH-dependent sulfite/nitrite reductase (BN5_1900/W6RF17) encoded by the *cio* gene cluster [25]. Although a role of this putative sulfite/nitrite reductase on cyanide assimilation has not been demonstrated, the jewelry wastewater also contains a small concentration of nitrite and cross-talk between nitrite and sulfite reductases, considering that they share similar mechanisms of catalysis, could be regarded.

The ferric uptake regulation (Fur) protein (BN5_0907/W6QZB3) was identified as a target of sRNA649 and sRNA258. Fur is a global transcription factor that regulates iron homeostasis, which has been proposed to have a role on keeping the iron pool during cyanide assimilation in the strain CECT5344 [55]. Iron uptake in bacteria is usually mediated by low molecular weight molecules called siderophores, which are secreted during iron limiting conditions [55]. An additional target of sRNA649 was a putative protein involved in siderophore biosynthesis encoded by the BN5_1295 gene (S2 Table). A target of sRNA649 was the regulatory protein GntR-like protein of *P*. *pseudoalcaligenes* CECT5344 (BN5_1352/W6QVD8). In a previous work, another GntR-like protein encoded by the BN5_1894 gene was disrupted, and the defective mutant strain showed a reduced growth rate on cyanide [24], suggesting that GntR proteins may participate in the regulation of the jewelry residue detoxification by the strain CECT5344. Other targets of sRNA649 were the alternative oxidase CioB component (BN5_1903/W6QVH5) and the methylenetetrahydrofolate reductase MetF (BN5_1909/W6QWY1). The relevance of methionine on cyanide degradation has not been demonstrated, but curiously other two sRNAs (sRNA184 and RNA649) have as predicted targets the MetN and MetF component involved in methionine metabolism.

From all sRNAs identified with a potential function in degradation of the cyanide-containing jewelry residue, sRNA222 and sRNA649 displayed the highest number of target genes, including 15 genes located in 8 gene clusters (Fig 5 and Table 2). The increased expression of most of these gene targets evidence that they are positively regulated by the corresponding sRNAs (Table 2).

In summary, in this work sRNAs have been identified highlighting that sRNA-mediated regulation is of relevance in cyanotrophic bacteria lifestyle, contributing to increase the efficiency of cyanide-containing industrial wastes bioremediation processes.

## Supporting information

S1 Table Oligonucleotides used in this work. (DOCX)

S2 Table Putative targets of *P*. *pseudoalcaligenes* CECT5344 sRNAs. (XLSX)
