# Supplementary material for: Putative small RNAs controlling detoxification of industrial cyanide-containing wastewaters by Pseudomonas pseudoalcaligenes CECT5344
Source: PLoS One. 2019 Feb 8;14(2):e0212032. doi: 10.1371/journal.pone.0212032 (PMC6368324; doi:10.1371/journal.pone.0212032)
Supplement: S1 Table — (DOCX) [file pone.0212032.s001.docx]

**Table S1. Oligonucleotides used in this work.**

| **Primer** | **Sequence (5’ 🡪 3’)** |
| --- | --- |
| RT-683F | tgccagtcagttaagccgttgcact |
| RT-683R | gttcgtcaccaccccctcctacttg |
| RT-455F | tcagcgcacgcggtaacggatagtg |
| RT-455R | cgccgcgatgctgttcaacctg |
| RT-655F | tggcagcgtcgcacagggtttga |
| RT-655R | cgatttggtttgcatgggcgggatt |
| RT-679F | ctattcggcagtgaagtttctaggc |
| RT-679R | gcaaattccttgggggatca |
| RT-184F | cggaaactgcaggacgcacttttgtccaggca |
| RT-184R | attcagtcggactcttcagattatctcctca |
| RT-222F | gcacgagtagtggaggtgctgtggaggagg |
| RT-222R | tcgctccggcgctgttccgagggtcgtcgc |
| RT-149F | gaagtggcggtagaaactgtcgaagaaatc |
| RT-149R | ccaatctggtgatgcagagctatggacgctg |
| RT-150F | gaggaaggcgaacaggcacaggcccaggccgagg |
| RT-150R | gccctgatgatgtacgttctggtgggcggcc |
| RT-312F | cgattgctggcacaacgacaatcatcatga |
| RT-312R | aactcaaaaggggcggtaggcatcgcccag |
| RT-649F | gcacagggctgggcaacctccctaccca |
| RT-649R | cggtgaccaggtcggttttggccggg |
| RT-601F | ctgtccagcgtgtccctgttcgtct |
| RT-601R | gcacatggattcagcgcaggaagc |
| RT-431F | gccagttaagcaggtaaaggtgagt |
| RT-431R | cttcaagcgggctgatgatt |
| RT-258F | aaatcaaggtacgcggtccacggta |
| RT-258R | ggaacgtactggtgtgcggcatct |
| RT-14F | ggcgacccgagggttaatgctgatcagataagttagtcac |
| RT-14R | ggctcctacgaaaagcgcccctctactgcaagtag |
| RT-511F | gcagtagagggggcgcttttcgtaggagcca |
| RT-511R | cggggtaatgtcagtcagttaagccgttgcactcg |
| RT-559F | acgctttcgtgcaacctgccgcagcaagcc |
| RT-559R | cgcgccgccgacattccgacagcacct |
| qRT-415F | gtgcttcgacgacggccatga |
| qRT-415R | ctgcgtcaggtattcggcccagagt |
| qRT-417F | gtaccgaccgcagcctgagccgtct |
| qRT-417R | ccggtgatgatcagcgatgcgtcct |
| qRT-550F | cggcgtcgacatgctggagatggat |
| qRT-550R | agtgcttgcagttcggcgaggctga |
| qRT-551F | cgtcgccctgaccgccctct |
| qRT-551R | gcataaccgaaggggctggccagat |
| qRT-554F | gaatgcctgcaagccaatgacgac |
| qRT-554R | gcagggcgacatggcggttg |
| qRT-570F | cggatagtggccttgcgtgacgaag |
| qRT-570R | cggccaactgacctgcgaggaagt |
| qRT-574F | ctctcgatcatcgcccgtaccca |
| qRT-574R | aaatccttgatcccgaccaggcaga |
| qRT-575F | tgcgatgcaatcattcccgaggct |
| qRT-575R | ggtcgtgctcggcctgacagttgac |
| qRT-577F | tgttgagccaccgacgtcagacgaa |
| qRT-577R | gcgtcaccagcccatgcttgagc |
| q-RT598F | gccaaatgccagaacgtcgccatc |
| q-RT598R | cggaacagatcgaccacgcctttga |
| q-RT607F | gctactcgcgctcggcctcggtatg |
| q-RT607R | attgcgcaccgtgaccatccaagtg |
| q-RT830F | cgcggcgaagatgaccaagacca |
| q-RR830R | ggtgcccgggttgtatttgtccagg |
| q-RT834F | gcacgcagttcaacgccaacgaaat |
| q-RT834R | gcgggccatgcacgaagaacaac |
| q-RT835F | ccagtggccaatctgcacccggatg |
| q-RT835R | agcagggcgttgtccaccaggctca |
| q-RT1092F | caagcaccatggcctgaccgacacc |
| q-RT1092R | aggaaacgacggatgtgcgcgatca |
| q-RT1094F | cgtgccggctccaatctctaccagg |
| q-RT1094R | agaggtaccagcggcggccatgat |
| q-RT1096F | tggcaccagcatcggcatcag |
| q-RT1096R | cgttgttcagctccttgcggatcac |
| q-RT1629F | cagcttcctgttcatgcgcgttgc |
| q-RT1629R | cagtttgccgcgaaagtcccagtcc |
| q-RT1631F | gcgttcatcgaggaaggcggtgag |
| q-RT1631R | cagatccatcacgtccagcgggttg |
| q-RT1632F | cgcgaagctggatggtggtgtc |
| q-RT1632R | ggagtgatcttgcgccgatgctgaa |
| q-RT1637F | gcgtgcgcgacaacaccaaccacta |
| q-RT1637R | gagacgctgcagcaggccgaacaac |
| q-RT1642F | cgagttgccgtgcgatctggatctc |
| q-RT1642R | ggcatcttcgaaccacaccgcttca |
| q-RT1900F | cgtgagcgattcgtggcacctgact |
| q-RT1900R | ggtgcgtgttgttgcgaacccagtg |
| q-RT1903F | cgcacgctcaatgacgaccc |
| q-RT1903R | cgatggaggcacgatgttgg |
| q-RT1909F | ccaaggggctgcatgtggaaatcac |
| q-RT1909R | agacaccggctggactggggatg |
| q-RT2264F | gtcgaaactcatcctgcgcggcaag |
| q-RT2264R | gcagacggcgctcggcgatctt |
| q-RT2265F | cgagcgcgacttcaagggcttctcc |
| q-RT2265R | ccaccgccctcgctgcctaagatgt |
| q-RT2268F | tggatgagcgcggcgacttcttctt |
| q-RT2268R | tctgcgcttggctgatgggtagtgg |
| q-RT2308F | ggcgacttcaaccacggcttcacct |
| q-RT2308R | cacccgctcgacgatgccctct |
| q-RT2309F | ctggtgctggcgggattgctga |
| q-RT2309R | ggcgtcgagcatgtccttgggaaag |
| q-RT2311F | gcttaccgcgctggcctctacgacc |
| q-RT2311R | cccgtgccctgcttgtcctgactca |
| q-RT3528F | tggcgctcagcacaatccatcgtca |
| q-RT3528R | ggtgggtcggtacggcgtggc |
| q-RT3532F | ctgcagctttggattcccggtgtgg |
| q-RT3532R | gtgcttgatgtgccgggcgaagac |
| q-RT3533F | ggaaggtcccctgcaatccattctc |
| q-RT3533R | ccactggtatcgccgaaagccaac |
| q-RTrpoBF | cgctggtgagcaagggggatcaggt |
| q-RTrpoBR | cgctcggacaggcagatggagtcttc |
